# Case report: High-grade endometrial stromal sarcoma with adrenal glands metastases: An unreported site of metastasis

**DOI:** 10.3389/fonc.2022.1058700

**Published:** 2022-11-23

**Authors:** Tao Zhang, Run-lin Feng, Si-fan Yin, Wen-bo Feng, Zhi-yuan Yin, Hao Wang, Chang-Xing Ke

**Affiliations:** ^1^ Department of Urology, The Second Affiliated Hospital of Kunming Medical University, Kunming, China; ^2^ Department of Pathology, The Second Affiliated Hospital of Kunming Medical University, Kunming, China; ^3^ Department of Nuclear Medicine, Sichuan Provincial People’s Hospital, University of Electronic Science and Technology of China, Chengdu, China

**Keywords:** endometrial stromal sarcoma, adrenal glands, diagnosis, treatment, clinical features

## Abstract

**Background:**

Endometrial stromal sarcoma is a relatively rare malignant tumor that derives from the malignant transformation of primitive uterine mesenchymal cells. It can lead to distant metastases. High-grade endometrial stromal sarcoma is extremely rare. The adrenal glands are an unreported site of metastasis.

**Case summary:**

A 71-year-old woman with a diagnosis of endometrial stromal sarcoma 30 months ago. After receiving treatment with radiotherapy and chemotherapy, the patient was kept asymptomatic during the follow-up until 2 years later, when she complained of dyspnea. Pulmonary and right adrenal gland metastases were detected by ^18^F-FDG PET/CT. The right upper lobe mass was diagnosed as a high-grade endometrial stromal sarcoma metastasis after postoperative pathology. Due to the patient’s high risk of surgery, as she had many underlying diseases, we performed adequate preoperative preparation. The physical examination revealed that a hard mass was palpable in the right renal area. The right adrenal mass was resected in our hospital. Immunohistochemistry showed ER (-), PR (-), CD10 (+), P16 (+), Ki-67 (50%). The final diagnosis on pathological examination was a high-grade ESS metastatic to the right adrenal gland. The patient continued treatment in other hospitals after surgical resection. After four months of postoperative follow-up, metastasis was detected again during a PET/CT examination at an outside hospital.

**Conclusion:**

Endometrial stromal sarcoma is rare, and the adrenal glands are an unreported site of metastasis. It has no specific clinical symptoms and mainly found for other reasons. The diagnosis still depends on pathology and immunohistochemistry. If there is no relevant past history, it is difficult to exclude a primary adrenal tumor.

## Introduction

Endometrial stromal sarcoma (ESS) is a rare malignant tumor of mesenchymal origin (1). Currently, there are four types of ESS (2, 3). It is a rare aggressive tumor, known to metastasize to lymph nodes, bones and lungs (2). There are no reports in the literature that ESS metastasizes to the adrenal glands. In this article, we retrospectively analyzed the clinical data of a patient with high-grade ESS metastasis to the right adrenal gland (2), which we cannot exclude as primary adrenal tumors before surgical resection. Therefore, we analyzed the clinical features of this disease in order to further improve our understanding of the disease and thus improve the diagnosis and treatment of this disease.

## Case introduction

A 71-year-old woman with diagnosis of ESS 30 months ago. After receiving treatment with radiotherapy and chemotherapy, the patient remained asymptomatic during the follow-up until 2 years later, when she complained of dyspnea.

**Figure 1:**
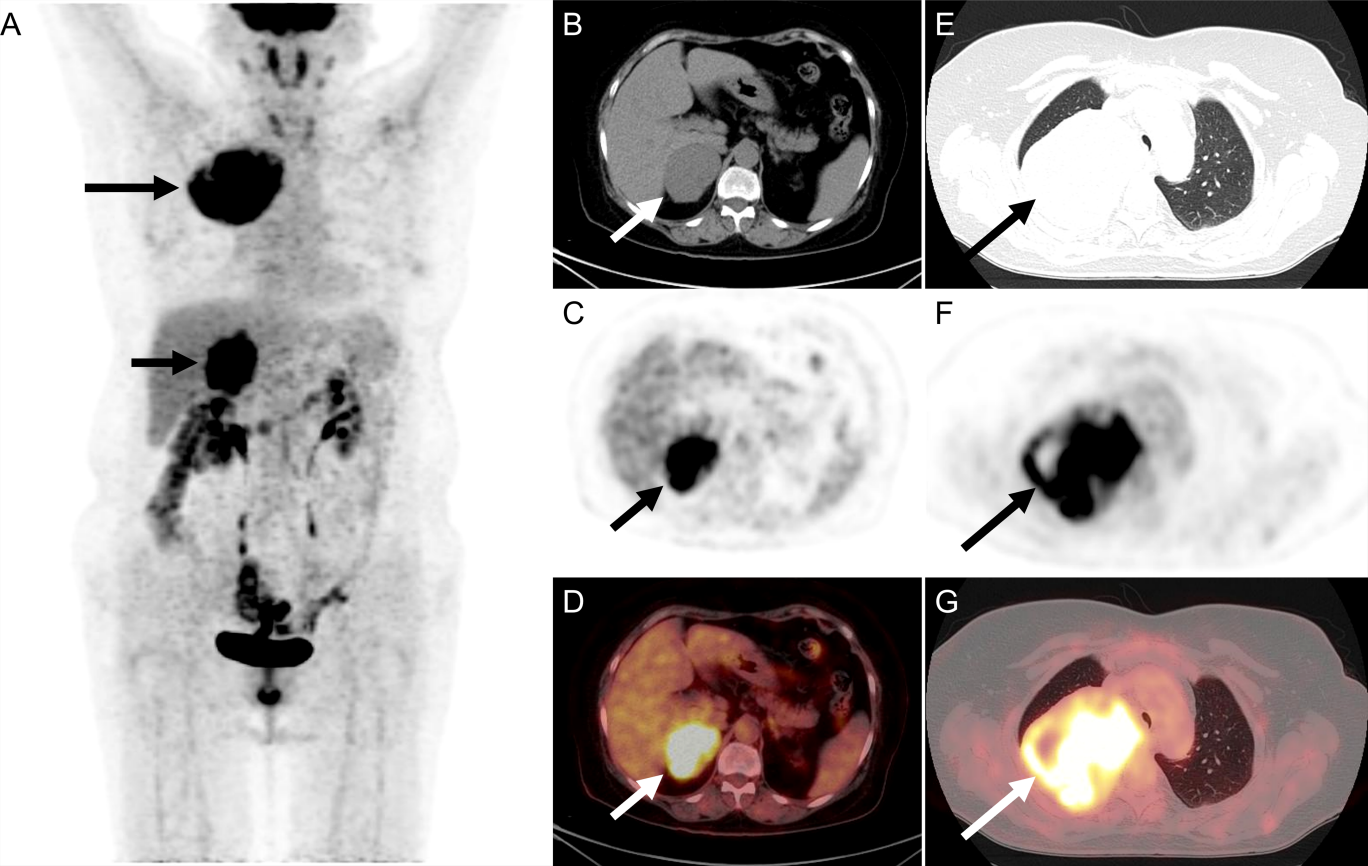
The ^18^F-FDG PET**/**CT of patient before operation. **(A)** The image revealed hypermetabolic lesions in the right thorax (long arrow) and the right upper abdomen (short arrow). **(B–D)** On the axial CT, PET, and fused PET/CT showed a large irregular lesion in the right lung upper lobe, measuring 8.4 cm×10.2 cm×8.1 cm showing intense ^18^F-FDG uptake with SUVmax of 13.4. **(E–G)** The axial CT, PET, and fused PET/CT revealed the increased activity corresponded to a slight hypodense right adrenal gland soft tissue mass, measuring 5.1 cm×6.3 cm×6.5 cm showing intense ^18^F-FDG uptake with SUVmax of 13.4.

^18^F-FDG PET/CT was performed to identify if there was a potential malignancy tumor. The maximum intensity projection image (**Figure 1A**) revealed hypermetabolic lesions in the right thorax ((long arrow) and the right upper abdomen (short arrow). On the axial CT (**Figure 1B**), PET (**Figure 1C**), and fused PET/CT (**Figure 1D**) showed a large irregular lesion in the right lung upper lobe, measuring 8.4 cm×10.2 cm×8.1 cm showing intense ^18^F-FDG uptake with SUVmax of 13.4. The axial CT (**Figure 1E**), PET (**Figure 1F**), and fused PET/CT (**Figure 1G**) revealed the increased activity corresponded to a slight hypodense right adrenal gland soft tissue mass, measuring 5.1 cm×6.3 cm×6.5 cm showing intense ^18^F-FDG uptake with SUVmax of 13.4. The patient underwent thoracoscopic resection of the right upper lobe at an outside hospital, and postoperative pathology diagnosed high-grade ESS metastasis to the right side of the lung. The patient had follow-up outside the hospital. After 2 months, the ultrasound revealed a right adrenal mass that was 9.1 × 6.5 cm. After 5 months, the ^18^F-FDG PET/CT revealed a right adrenal mass that was 11.4 cm × 11.0 cm × 10.8 cm. After a period of recuperation at home, she was came to our hospital for further treatment.

The past medical history was significant for hypertension and coronary artery disease. Coronary stenting was performed, and she was usually treated with oral aspirin anticoagulation and avastatin lipid regulation. Physical examination revealed a palpable and hard mass, in the right area of her kidney. The levels of CA-125 were normal (17.49 U/mL, normal reference values: 0-35 U/mL). The levels of CA-199 were abnormal (36.74 U/mL, normal reference values: 0-35 U/mL). Plasma ACTH, angiotensin and renin concentrations were within the normal range. Other laboratory tests showed no significant abnormalities.

**Figure 2:**
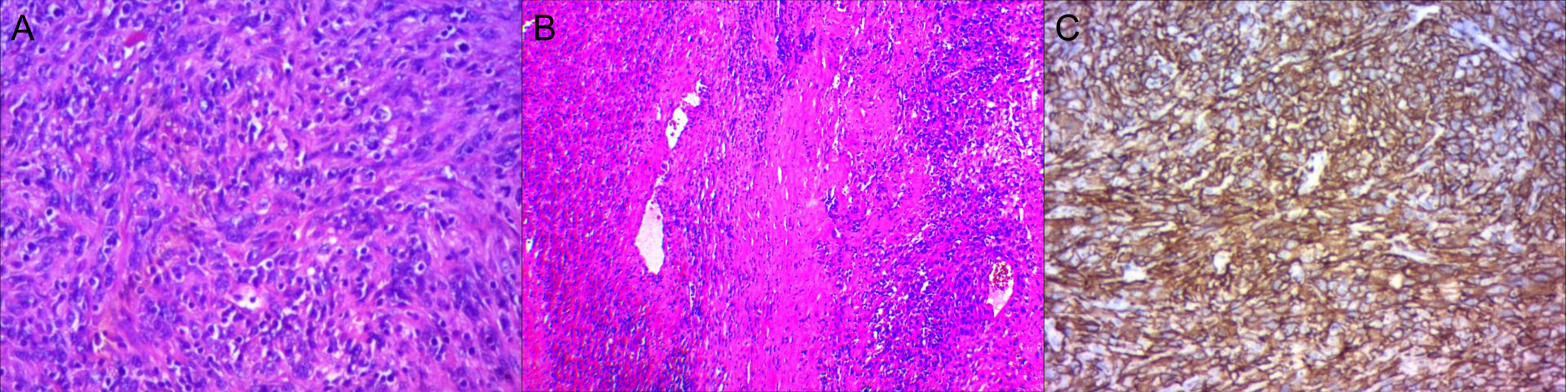
High-grade Endometrial Stromal Sarcoma in the right adrenal gland. **(A, B)** The tumor cells were seen in a swirling flow structure. **(C)** CD10 (+).

With adequate preoperative preparation, an open right adrenal tumor resection was performed. The intraoperative exploration revealed a cystic solid mass with a size of about 11.0 cm×10.5 cm. In a pathological view, the lesion was a round, dark red in color, and surrounded by a thick membrane and adhered to the surrounding tissues. On histopathological examination, the tumor cells were seen arranged in a swirling flow structure (**Figures 2A, B**). Immunohistochemistry showed: ER (-), PR (-), CD10 (+) (**Figure 2C**), P16 (+), Ki-67 (50%). The postoperative pathological diagnosis confirmed the high-grade ESS with right adrenal gland metastases. After 4 months of postoperative follow-up, metastasis was detected again during a ^18^F-FDG PET/CT examination at an outside hospital. The PET/CT of the outside hospital showed thickening of the left adrenal gland union with increased metabolism.

**Figure 3:**
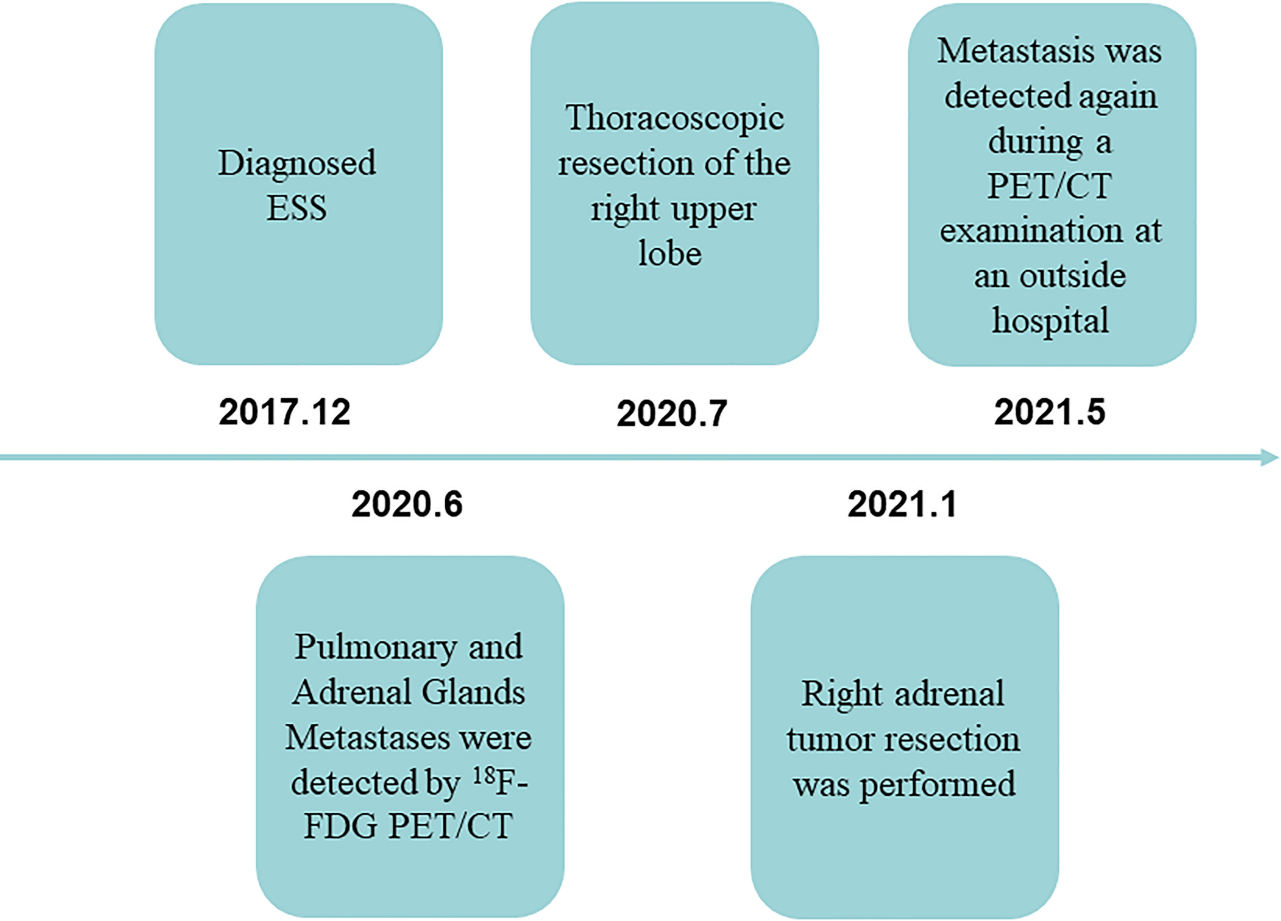
Timeline.

Timeline: Diagnosed ESS for 30 months—Pulmonary and Adrenal Glands Metastases were detected by ^18^F-FDG PET/CT before 6 months—She had undergone thoracoscopic right upper lobe resection 5 months ago, and postoperative pathology diagnosed high-grade ESS metastasis in the right lung—Right adrenal tumor resection was performed—After 4 months of postoperative follow-up, metastasis was detected again during a ^18^F-FDG PET/CT examination at an outside hospital (**Figure 3**).

## Related literature learning

Endometrial stromal sarcoma (ESS) is a relatively rare malignant tumor, which accounts for 0.2% of all uterine malignancies. The annual incidence of ESS is 1-2 per million women (1). There are four types of ESS: endometrial stromal nodule, undifferentiated uterine sarcoma, high-grade ESS and low-grade ESS (2, 3).

Chromosome translocations and rearrangements are common events in ESS and reflect distinct mechanisms of occurrence (4). Low-grade ESS is most commonly characterized by JAZF1-SUZ12 fusions followed by rearrangements involving PHF1 and multiple fusion partners (including JAZF1, EPC1, EPC2 and MEAF6). Furthermore, YWHAE gene rearrangement may be associated with high-grade ESS (5). However, genetic testing was not performed on our patients. The mean age of patients was 50 years and ranged from 28 to 67 years old. In addition to occurring around the uterus, it can also occur in the ovaries, rectal wall, pelvic peritoneum, and vagina. ESS occurring outside the uterus may be associated with endometriosis. However, it may also be unrelated to endometriosis (6). Clinical symptoms are variable, non-specific, and largely depend upon the location of ESS. When ESS is located in the uterus, abnormal vaginal bleeding and abdominal pain are the most common symptoms (3, 6).

Owing to its aggressive behavior, ESS can metastasize to lymph nodes, lungs, and bones, but it is relatively rare (2). Meanwhile, the incidence of high-grade ESS is extremely low. There was no report of adrenal gland metastases. In this case, we reported a very rare case of ESS with simultaneous metastasis to the right adrenal gland and right lung. The patient had been previously diagnosed with ESS and had received several cycles of chemotherapy (due to the absence of surgical guidelines). PET/CT imaging revealed occupations of the right lung and right adrenal gland (both considered metastases). The lung metastasis was confirmed. Surgical resection for pulmonary metastasis was performed. The exact details of the surgery, which were performed at an outside hospital, were not known.Adrenal occupancy was seen to be progressively larger on multiple postoperative reviews. So she was seen at our hospital for further treatment. The patient who has a history of ESS would be highly considered as a possible case of adrenal gland metastasis.In the literature, there was no reported ESS case that had metastasized to the adrenal gland (2). Primary adrenal malignancies could not be excluded in this patient.

ESS lacks specific clinical manifestations and imaging features. Microscopic examination showed that the tumor cells are arranged in a swirling flow structure and are located around the small arterial network of the hyaline wall (7). Necrosis may be seen, but is less common in low-grade ESS (7). High-grade ESS cells have irregular nuclear outlines, lack nucleoli, and have a high nuclear division index (but it is meaningless to distinguish between low-level and high-level ESS). High-grade ESS is the most malignant of the four classifications. Low-grade ESS characteristically shows diffuse positivity for CD10, ER, and PR, while high-grade ESS typically shows absent or only focal and weak staining for CD10, ER and PR (8, 9). In this case, macroscopically, the lesion was a gray-brown mass with an envelope, measuring about 12cm×12cm×6.5cm. Tumor cells mainly arranged in a swirling pattern. Immunohistochemistry: ER (-), PR (-), CD10 (+), P16 (+), Ki-67 (50%). The final diagnosis was that high-grade ESS metastasized to the right adrenal gland.

If there is no history of endometriosis, diagnosing ESS remains challenging. Some patients have no history of endometriosis (6, 10). Patients with high-grade ESS need to be distinguished from those with the following other diseases. 1) Undifferentiated uterine sarcoma: This is a high-grade sarcoma that is extremely rare and lacks a specific line of differentiation. The morphology and immunohistochemistry lack evidence of differentiation. Therefore, the prognosis of the disease is poor (11–13). Chemotherapy, hormonal therapy, and radiotherapy are the existing treatment options currently available. 2) Leiomyosarcoma: Leiomyosarcoma is a malignant mesenchymal tumor that originates from the smooth muscle. The incidence increases with age. Leiomyosarcoma can occur in the gastrointestinal tract and uterus. The adrenal glands can be seen (14). The clinical presentation is not specific. It is commonly discovered incidentally. On microscopic analysis, tumor cells show cytological atypia with cigar-shaped nuclei and abundant eosinophilic and fibrillar cytoplasm. When the cells were well differentiated, the cells were seen to be smooth muscle-like (15). The diagnosis is based on the demonstration of desmin, smooth muscle actin, actin HHF-35, and h-caldesmon. The main treatment method is surgery-assisted chemotherapy (14).

Because of the low incidence, there has been no consensus on the standard treatment of high-grade ESS (15). For ESS, complete surgery is the main treatment. Hormone therapy is recommended for patients with low-grade ESS. Chemotherapy with gemcitabine and docetaxel is used in high-grade ESS (8). Immunotherapy of high-grade ESS is still under investigation (16). High-grade ESS, with a median survival time of 11-23 months, is more aggressive and has a lower prognosis than low-grade ESS (7, 16, 17). The patient underwent surgery at our hospital, while the continuation phase was given in another hospital. After 4 months of postoperative follow-up, metastasis was detected again during a PET/CT examination at an outside hospital.

In conclusion, ESS is a rare malignancy, and the symptoms differ greatly depending on the location. High-grade ESS can metastasize throughout the lungs, lymph nodes, and bone. High-grade ESS is particularly rare and this is the first report of a metastasis to the adrenal glands. The diagnosis of metastatic ESS should be considered when the patient has a history of ESS and presents with a mass at a distant site. However, the diagnosis depends on the pathology and needs to be differentiated from other diseases. Surgical resection is used as the first treatment of choice, and high-grade ESS needs to be followed by concurrent chemotherapy and requires follow-up after surgery.

## Data availability statement

The original contributions presented in the study are included in the article/supplementary material. Further inquiries can be directed to the corresponding authors.

## Ethics statement

Written informed consent was obtained from the individual(s) for the publication of any potentially identifiable images or data included in this article.

## Author contributions

TZ, R-LF and S-FY collected data and drafted the manuscript. R-LF and HW provided figures and pathology results and drafted the manuscript. W-BF and Z-YY review the manuscript. HW and C-XK edited the manuscript and critically revised the draft. All authors contributed to the article and approved the submitted version.

## Conflict of interest

The authors declare that the research was conducted in the absence of any commercial or financial relationships that could be construed as a potential conflict of interest.

## Publisher’s note

All claims expressed in this article are solely those of the authors and do not necessarily represent those of their affiliated organizations, or those of the publisher, the editors and the reviewers. Any product that may be evaluated in this article, or claim that may be made by its manufacturer, is not guaranteed or endorsed by the publisher.
